# The Role of PLAG1 in Mouse Brain Development and Neurogenesis

**DOI:** 10.1007/s12035-024-03943-w

**Published:** 2024-01-19

**Authors:** Jemma G. Gasperoni, Stephanie C. Tran, Sylvia V. H. Grommen, Bert De Groef, Sebastian Dworkin

**Affiliations:** 1 Department of Microbiology, Anatomy, Physiology and Pharmacology, School of Agriculture, Biomedicine and Environment, La Trobe University, Bundoora, Victoria 3086 Australia; 2 Department of Biology, KU Leuven, B3000 Leuven, Belgium

**Keywords:** Plag1, Mouse model, Neurobiology, Neurosphere, Stem/progenitor cells

## Abstract

The pleomorphic adenoma gene 1 (*Plag1*) is a transcription factor involved in the regulation of growth and cellular proliferation. Here, we report the spatial distribution and functional implications of PLAG1 expression in the adult mouse brain. We identified Plag1 promoter-dependent β-galactosidase expression in various brain structures, including the hippocampus, cortex, choroid plexus, subcommisural organ, ependymal cells lining the third ventricle, medial and lateral habenulae and amygdala. We noted striking spatial-restriction of PLAG1 within the cornu ammonis (CA1) region of the hippocampus and layer-specific cortical expression, with abundant expression noted in all layers except layer 5. Furthermore, our study delved into the role of PLAG1 in neurodevelopment, focusing on its impact on neural stem/progenitor cell proliferation. Loss of Plag1 resulted in reduced proliferation and decreased production of neocortical progenitors *in vivo*, although *ex vivo* neurosphere experiments revealed no cell-intrinsic defects in the proliferative or neurogenic capacity of Plag1-deficient neural progenitors. Lastly, we explored potential target genes of PLAG1 in the cortex, identifying that Neurogenin 2 (*Ngn2*) was significantly downregulated in Plag1-deficient mice. In summary, our study provides novel insights into the spatial distribution of PLAG1 expression in the adult mouse brain and its potential role in neurodevelopment. These findings expand our understanding of the functional significance of PLAG1 within the brain, with potential implications for neurodevelopmental disorders and therapeutic interventions.

**Supplementary Information:**

The online version contains supplementary material available at 10.1007/s12035-024-03943-w.

## Introduction

The pleomorphic adenoma gene 1 (PLAG1) is a zinc finger transcription factor involved in multiple cellular processes, including cell growth, differentiation and embryonic development. PLAG1 was initially characterised as an oncogene, first discovered to be upregulated in pleomorphic adenoma, a benign tumour of the salivary glands. Since then, overexpression of *PLAG1* has been identified in several cancer types including rhabdomyosarcoma [1], uterine myosarcoma [2], lipoblastoma [3] and myoepithelioma [4]. In rhabdomyosarcoma, PLAG1 was shown to upregulate insulin-like growth factor 2 (*Igf2*), a known target gene [1], alter alpha serine/threonine kinase (AKT) and mitogen-activated protein kinase (MAPK) pathways and positively regulate proliferation and survival of rhabdomyosarcoma cells [5]. PLAG1 also drives cancer metastasis via its transcriptional regulation of isocitrate dehydrogenase (NADP)-specific glutamate dehydrogenase 1 (*Gdh1*), mediating anti-anoikis and pro-metastatic signalling within cancer cells [6]. Together, these studies had implicated *Plag1* as a key gene in mediating pro-survival and pro-proliferation cellular pathways.

In addition to known roles in cancer, PLAG1 is also a critical developmental factor. PLAG1 is expressed in multiple embryonic cell lineages, with expression declining in most tissues postnatally [7–10]. Mice with a germline deletion of *Plag1* (*Plag1*^*−/−*^) presented with pronounced growth retardation, first detectable at embryonic day 11.5 (E11.5) and continuing throughout foetal development; by post-natal day 21 *Plag1*^*−/−*^ mice weigh 50% less than wildtype (WT) littermates [8]. Concomitant with a critical role in embryogenesis and reproduction, *Plag1*^*−/−*^ male mice present with significant defects in fertility due to lowered daily sperm counts and reduced sperm motility [11] and also abnormal morphology and defective coiling of the epididymis [12]. These findings indicate a key role in developmental homeostatic regulation of cellular growth, proliferation and tissue maintenance.

Although PLAG1 expression decreases significantly in numerous tissues post-natally, persistent PLAG1 expression in adult has been confirmed in testis and pituitary [11, 13], ovary [14] and particularly in neurons of the brain [13], suggesting that PLAG1 may also regulate tissue-specific post-developmental organ homeostasis. Experiments to characterise possible roles in adult behaviour suggested a defect in freezing and startle response [15], consistent with robust *Plag1* expression in adult amygdala, a region crucial for fear conditioning [16]. Abundant *Plag1* expression within the hypothalamus and pituitary gland suggested a regulatory role in the hypothalamo-pituitary system in males [13]. However, gene expression data have failed to reveal any functional changes in the hypothalamo-pituitary system [13] indicating that the effects of PLAG1 on reproduction act downstream of this system.

In the developing brain, PLAG1 is expressed as early as E9.5 in the dorsal telencephalon, diencephalon and midbrain and at relatively lower levels in the neural tube and hindbrain [7]. At E12.5, strong PLAG1 expression is observed in cortical progenitors, with lower levels of expression also found in the ventricular zone (VZ) of the lateral and medial ganglionic eminences [7, 17]. Loss of *Plag1* at this timepoint both in *Plag1*^*−/−*^ mice [17] and via in vitro short-hairpin ribonucleic acid (shRNA) knockdown approaches at E11.5 [18] reduces the number of proliferating neocortical progenitors (NPC), but not the total number of cells, indicating a role for *Plag1* in driving NPC differentiation. These studies suggest that the role of PLAG1 goes well beyond the regulation of proliferation; however, the precise mechanisms of PLAG1-mediated regulation of embryogenesis and tissue maintenance remain elusive. Moreover, the expression and function of PLAG1 in other regions of the brain remains unexplored, and the functional role in maintenance and differentiation of neural stem/progenitor cells (NSPCs) at E14.5 has not yet been characterised.

A significant roadblock to identifying *Plag1*-dependent genetic mechanisms arises from the widespread presence of the consensus PLAG1-binding sequence, found within promoter or enhancer regions of approximately 25% of all genes [19]. Whilst insulin-like growth factor 2 (*Igf2*) is an experimentally validated gene across numerous developmental and cancer contexts [20–26], it remains unclear if PLAG1 regulates *Igf2* expression in the embryonic cortex [17]. Moreover, the identity of other true target genes that operate within the brain remains unknown. To address this, a recent study characterised the transcriptome of *Plag1*-inhibited NSPCs to identify potential target genes through RNA-sequencing (RNA-SEQ) following transient inhibition of PLAG1 in vivo [18]. Although several putative candidate targets were identified, these are yet to be experimentally validated; interestingly, however, gene ontology analysis from the RNA-seq dataset indicated “nervous system development”, “synaptic”, “neurogenesis” and “behaviour” terms were strongly represented in the down-regulated genes [18]. These results are indicative of a role for *Plag1* in neural establishment and homeostasis.

Here, we performed a comprehensive characterisation of *Plag1* expression throughout the adult mouse brain, and additionally, investigated the consequences of PLAG1 deficiency during the final stages of embryogenesis and neural stem/progenitor cell proliferation and differentiation within the brain at E14.5.

## Methods

### Genotyping

*Plag1* knockout (KO) mice (*Plag1*^*−/−*^) were a kind gift from Prof. Wim Van de Ven, Laboratory for Molecular Oncology, Center for Human Genetics, KU Leuven, Belgium [8]. Ear or tail clips were used for PCR genotyping as previously described [8] using the following primers: 5′-ATGGCCACTGTCATTCCTGGTGATTTGTCA-3′ and 5′-CCTGTGTGTACCACCATGTGTCTCCGGACA-3′ to detect the WT *Plag1* allele and 5′-GCATCGAGCTGGGTAATAAGCGTTGGCAAT-3′ and 5′-ACACCAGACCAACTGGTAATGGTAGCGAC-3′ to detect the *lacZ* reporter gene.

### Animals

All mice were housed in ventilated cages under a standard 12-h light-dark cycle with *ad libitum* access to food and water.

In *Plag1*^*−/−*^ mice, the entire *Plag1* coding sequence has been replaced by the *lacZ* reporter encoding β-galactosidase [22]. This allows for spatiotemporally restricted expression of β-galactosidase in place of PLAG1 to be detected using histology, serving as a *de novo* marker of *Plag1* promoter activity. Throughout the text, for convenience, we use the phrase “*Plag1* expression” to indicate this.

### Tissue Fixation and Histological Preparation

Adult mice were anaesthetised via intraperitoneal injection with Lethabarb (90 mg/kg). The mice were then transcardially perfused using 0.05 M phosphate buffer (PB; 1 M Na_2_HPO_4_.2H_2_O, 1 M NaH_2_PO_4_.2H_2_O, pH 7.4), followed by perfusion with 4% (w/v) paraformaldehyde (PFA) in 0.1 M PB. Perfused brains were then removed, immediately placed in 4% PFA in 0.1 M PB and fixed for 4 h at 4 °C. Next, the tissues were cryoprotected, frozen and stored at −80 °C.

For embryonic tissue collection, pregnant dams were euthanised using CO_2_ at embryonic day (E) 14.5. The embryo heads were dissected and immediately placed into 4% PFA in phosphate-buffered saline (PBS), fixed for 24 h at 4 °C before cryoprotection and cryomold embedding.

### Combined X-Gal and Immunohistochemistry

X-gal staining on frozen adult brain sections (10 μm) and immunohistochemistry was conducted as described previously [11]. Nuclear Fast Red (NFR; 0.1% [w/v] aluminium sulphate) was used to counterstain X-gal slides. Primary antibodies used in this study were anti-NeuN (ab177487; Abcam), anti-Ki67 ab15580, anti-activated Caspase3 ab2302 (Abcam), anti-eomesodermin (EOMES) 14-4875-82 (Abcam), anti-Pax6 MA1109 (Thermo Fisher Scientific) anti-Tuj1 (# 14-4510-82; Invitrogen) and anti-GFAP (Z0334; Agilent Dako). Secondary antibodies used were goat anti-rabbit IgG H&L (ab6720; Abcam), donkey anti-rabbit IgG H&L Alexa Fluor 594 (R37119; Life Technologies), goat anti-rabbit 488 (A11008) and goat anti-mouse IgG H&L Alexa Fluor 555 (A32727; Thermo Fisher Scientific). Streptavidin-horse radish peroxidase (ab7403; Abcam) was used for subsequent visualisation. Imaging was conducted using an Olympus BX53 microscope attached to a DP73 camera. All image analysis was performed using ImageJ software (National Institute of Health, Bethesda, MD, USA; http://rsb.info.nih.gov/ij/download.html

### Normalising Cell Count for Reduced Brain Size

To account for smaller brain size of *Plag1*^−*/*−^ mice, 4′,6-diamidino-2-phenylindole (DAPI)+ cells were counted in four distinct regions of the cortex (a minimum of 4 images per embryo) in all wild-type (WT) and *Plag1*^−*/*−^ mice analysed in this study. These counts were then used to normalise and account for different brain size between genotypes.

### Embryonic NSPC Isolation and Culture

Embryonic NSPC extraction, culture and passaging and experimental protocols for cumulative cell number and neurosphere survival assays were all performed as described previously [27].

### Single-Cell Culture in Terasaki Wells

This protocol was as described in [27], with the following modifications. *Plag1*^−*/*−^ and WT NSPCs were plated in ultra-low volume Terasaki wells at a density of 1–3 cells per well (two Terasaki plates of 60 wells per culture per embryo; *n* = 3 separate individual mice per genotype) to assess clonal self-renewal potential.

### Differentiation Assay

This protocol was slightly adapted from [27], with respect only to the antibody used to identify newly-differentiated neurons (Tuj-1) used within the present study. Briefly, *Plag1*^−*/*−^ and WT neurospheres were cultured in 5% foetal calf serum (FCS) in neurosphere basal medium, comprising equal volumes of Dulbecco’s modified Eagle’s medium [DMEM] and F12, 4 μg/ml heparin and 100 μg/ml penicillin/streptomycin, for 7 days to induce differentiation. Differentiated cells were stained with neuronal (Tuj1) and astrocyte (Glial Fibrillary Acidic Protein; GFAP) antibodies to quantitate relative percentages of differentiated neural cell types. To quantitate the relative percentages of differentiated cell types generated, a minimum of five different fields were selected for counting per culture, experiment performed in duplicate.

### Quantitative Polymerase Chain Reaction

For quantitative polymerase chain reaction (qPCR), the cortex was dissected from E14.5 embryos (*n* = 3–5 for each of WT and *Plag1*^−*/*−^). β-actin (*Actb*) was used as the reference gene. qPCR data were processed, and fold changes presented using LinRegPCR v.2017.0 software [28]. Unpaired *t*-tests were performed using GraphPad Prism 9 (GraphPad Software, La Jolla, CA, USA) to compare average normalised mRNA expression levels. Primer sequences are listed in Supplementary Fig. S1.

### Statistical Analysis

Unless otherwise stated, results are expressed as mean ± standard error (SEM). Data were analysed either by Student’s unpaired *t*-test or one-way analysis of variance (ANOVA), with post-hoc Tukey’s multiple comparison test, as indicated in the text. *p* < 0.05 was considered to be statistically significant.

## Results

### PLAG1 Is Expressed Within the Adult Hippocampus

**Fig. 1:**
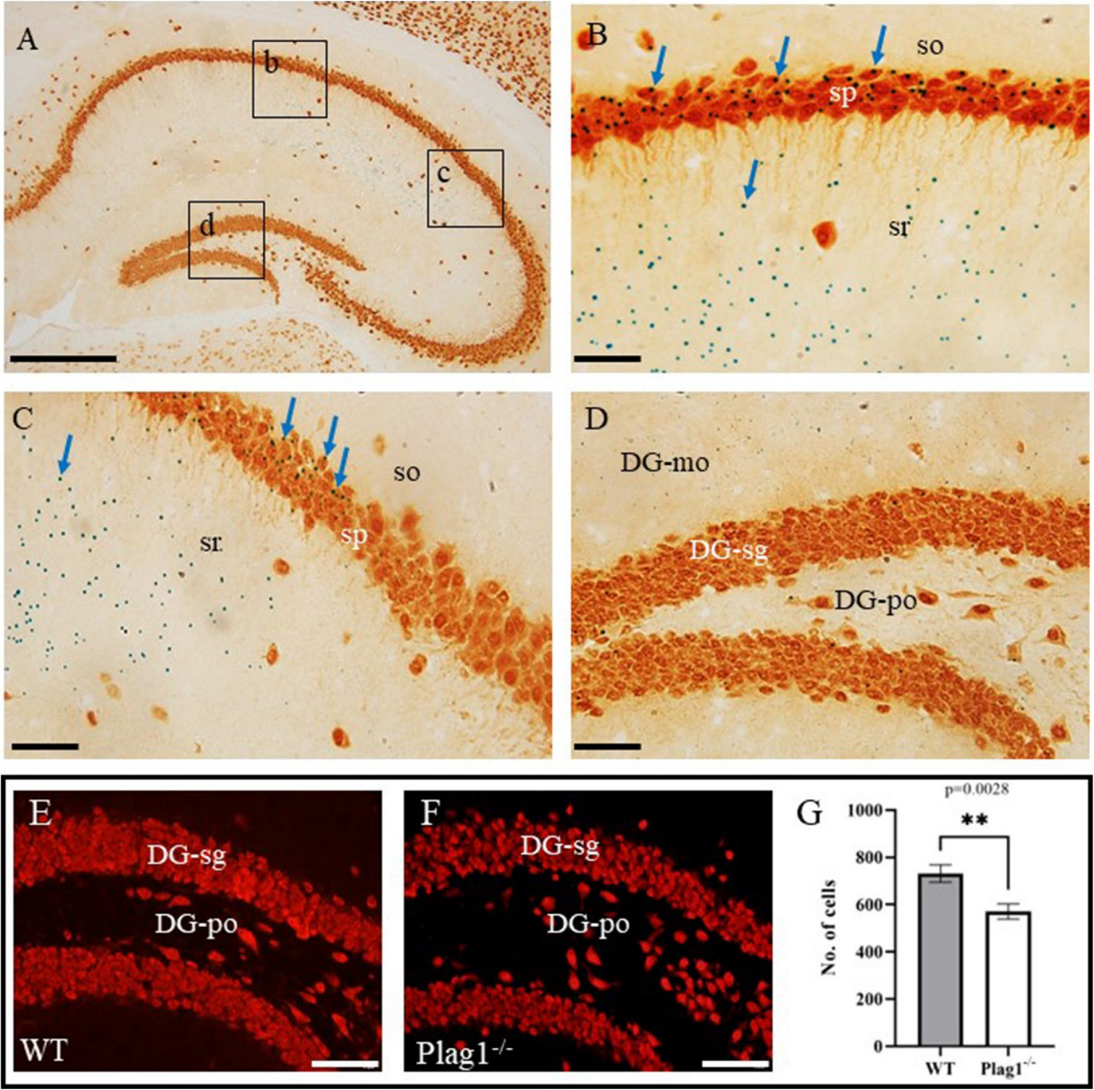
β-galactosidase expression driven from the *Plag1* locus in adult *Plag1*^−*/*−^ hippocampus and cortex. Combined X-gal (blue punctate staining; arrows) and immunohistochemical staining (anti-NeuN) in the hippocampus (**A**); stratum pyramidale (sp) and stratum radiatum (sr) in the CA1 region of the hippocampus (**B**); stratum pyramidale and stratum radiatum in the CA2 region of the hippocampus (**C**) and the molecular layer (DG-mo), granule layer (DG-sg) and polymorph layer (DG-po) of the DG (**D**). Labels (b–d) in **A** correspond to **B**, **C** and **D**, respectively. Representative images of NeuN expression within the DG region of the hippocampus in male *Plag1* WT (**E**) and KO (**F**). Number of neurons in the DG region of the hippocampus in male WT and Plag1^−/−^ mice (**G**), **p = 0.0028 (unpaired *t*-test). Error bars represent the standard error of the mean (*n* = 3 per genotype). DG-mo, molecular layer of dentate gyrus; DG-po, polymorph layer of the dentate gyrus; DG-sg, granule layer of the dentate gyrus; so, stratum oriens; sp, stratum pyramidale; sr, stratum radiatum. All images are representative of *n* = 3 individual animals. Scale bar = 500 μM (**A**) and 50 μM (**B**–**F**)

As previous analyses of PLAG1 expression in the adult brain focussed only on expression in the hypothalamo-pituitary system [13], we extended these analyses to investigate *Plag1* expression in regions of the brain not previously characterised. Within the hippocampus, strict localisation of *Plag1* expression was observed within the cornu ammonis (CA1) region (Fig. 1), where abundant expression was seen in the stratum pyramidale and stratum radiatum although expression was largely absent in the stratum oriens (Fig. 1A–D). Similarly, sparse expression was found in the dentate gyrus (DG). In brains from male *Plag1*^−*/*−^ mice, the number of cells positive for expression of *NeuN* in the DG region (570.8 ± 31.98) was significantly lower in comparison to brains from male WT littermates (731.4 ± 37.17; *p* = 0.0028; Fig. 1E–G). Surprisingly, this decrease in DG neurons of *Plag1*^−*/*−^ mice was not consistent throughout the entire hippocampus; in the CA1 region there was no significant difference in neuron number between WT (278.1 ± 7.66) and *Plag1*^−*/*−^ males (260.4 ± 18.54). These data indicate selective specificity of neuronal PLAG1 expression within the hippocampi of *Plag1*^−*/*−^ mice.

### Strict Spatial Localisation of PLAG1 Expression in the Adult Cortex

**Fig. 2:**
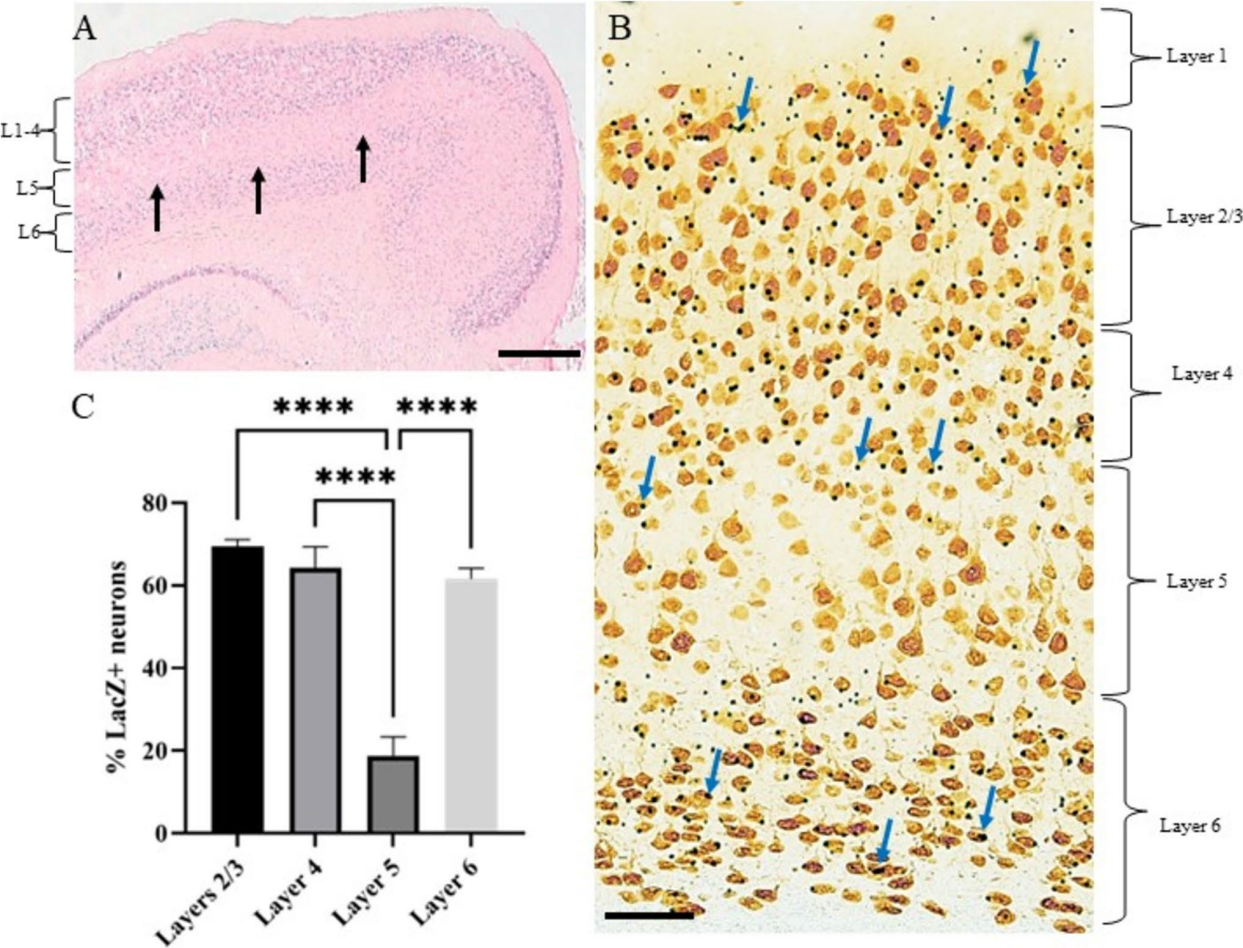
β-galactosidase expression driven from the *Plag1* locus in the cortical layers of adult *Plag1*^−*/*−^ mouse brain. Combined X-gal (blue punctate staining; arrows) and nuclear fast red (NFR) staining in the cortex of *Plag1*^−*/*−^ mice, showing a clear sparing of Plag1 expression within layer 5 (L5; black arrows) of cortical neurons, despite strong expression present in layers 1–4 (L1–4) and layer 6 (L6) (**A**). Higher-power magnification of cortical Plag1 expression using combined X-gal and anti-NeuN immunohistochemistry highlights spatially-restricted cortical layer localisation of PLAG1 (**B**). Quantitation of the proportion of LacZ+ neurons within each layer of the cortex shows that abundant *Plag1* expression is present in layers 2/3, 4 and 6 with comparatively sparse, significantly reduced expression in layer 5 (**C**). Cortical images and quantitation are representative of *n* = 3 individual animals. Values are expressed as mean ± standard error. ****p < 0.0001; for one-way ANOVA with post-hoc Tukey’s multiple comparison test. Scale bar = 500 μM (**A**) and 50 μM (**B**)

β-galactosidase expression driven from the *Plag1* locus was detected widely throughout the cortical layers of adult *Plag1*^−*/*−^ mice (Fig. 2A–C). Intriguingly, expression patterns within the disparate cortical layers showed that PLAG1 localisation was strictly spatially regulated, and one-way analysis of variance (1-way ANOVA) statistical analysis indicated significant differences in neuronal number throughout these layers. PLAG1 expression was abundant in layer 1; however, the precise number of cells was difficult to accurately quantify given the overall sparse number of neurons present in this layer. A high proportion of neurons within layer 2/3 presented with strong PLAG1 expression (69.51 ± 1.60%), and similar expression patterns were noted in layer 4 (64.21 ± 5.13%). There was notably lower expression found in layer 5 (18.79 ± 4.56%), although layer 6, harbouring the earliest-formed neurons, also presented with strong neuronal PLAG1 expression (61.62 ± 2.57%). Taken together, PLAG1 expression within the cortex does not correlate precisely with the temporal developmental origins of the cortical layers. Nonetheless, the overall striking regionalised expression patterns of *Plag1* within the cortex suggest highly specialised signalling and/or functional roles within the adult brain.

### Extra-cortical PLAG1 Expression in Adult Mouse Brain

**Fig. 3:**
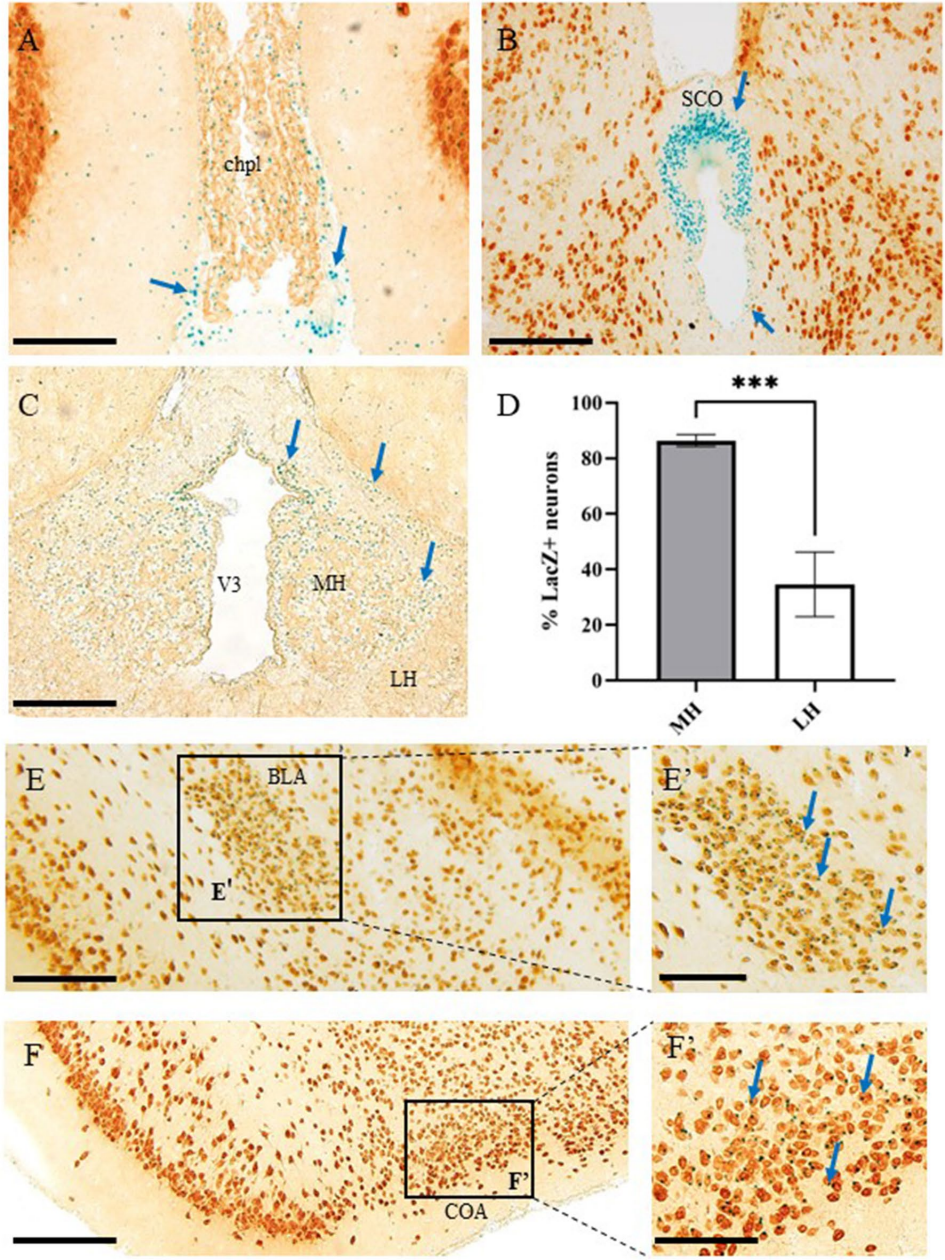
Extra-cortical localisation of β-galactosidase expression driven from the *Plag1* locus in adult *Plag1*^−*/*−^ mice brain. Combined X-gal (blue punctate staining; arrows) and immunohistochemical staining (anti-NeuN/anti-CD31) showing expression within the ventral choroid plexus (chpl; **A**), subcommissural organ (SCO; **B**), medial (MH) and lateral (LH) habenula and cells lining the third ventricle (V3; **C**). The MH comprises a significantly greater number of Plag1+ cells than the LH. Error bars represent the standard error of the mean; ***p = 0009 (unpaired *t*-test; **D**; *n* = 3 individual animals). Combined X-gal and immunohistochemical staining (anti-NeuN) in amygdala (**E**, **F**). X-gal+ cells are very strongly concentrated within the basolateral amygdala nucleus (BLA; **E-**E′) and the cortical amygdala area (COA; **F-**F′). Higher-magnification images of boxed regions in **E**, **F** (E′, F′) highlight regions of strong X-gal staining within the BLA (E′) and COA (F′), respectively. All images are representative of *n* = 3 individual animals. Scale bar = 400 μM (**A**–**C**, **E**, **F**), 250 μM (E′) and 200 μM (F′)

In order to extend our analyses of non-cortical PLAG1 expression, we explored the PLAG1 profile within deeper brain structures and have characterised numerous novel regions of PLAG1 expression. Firstly, our data show that PLAG1 is expressed within the choroid plexus (Fig. 3A), localised preferentially to the ventral-most region of this structure. Striking PLAG1 expression was also detected in the subcommissural organ (SCO), where PLAG1^+^ cells were localised in the subnuclear, supranuclear and intermediate regions, with exclusion zones in the subapical region and apical cell pole (Fig. 3B). We also noted strong expression in the ependymal cells lining the third ventricle, alongside marked expression within the medial (MH) and lateral habenulae (LH; Fig. 3C). Quantitative analysis indicated that the MH comprises a significantly greater number of Plag1+ cells than the LH (86.41 % ± 2.14 relative to 34.58% ± 11.65; ***p = 0009 unpaired *t*-test; Fig. 3D). Lastly, we also determined regionally localised expression patterns in the amygdala (Fig. 3E, F). Specifically, we identified abundant X-gal staining in the basolateral amygdala (BLA) and the cortical amygdala area (COA), but sparse expression in the piriform area of the amygdala (Fig. 3E, F). Taken together, our data clearly demonstrate regionalised, specific areas of abundant PLAG1-expression, which potentially correlate with site-specific roles in neuronal development and function.

### PLAG1 Is Required for Cell Proliferation in the Developing Cortex

**Fig. 4:**
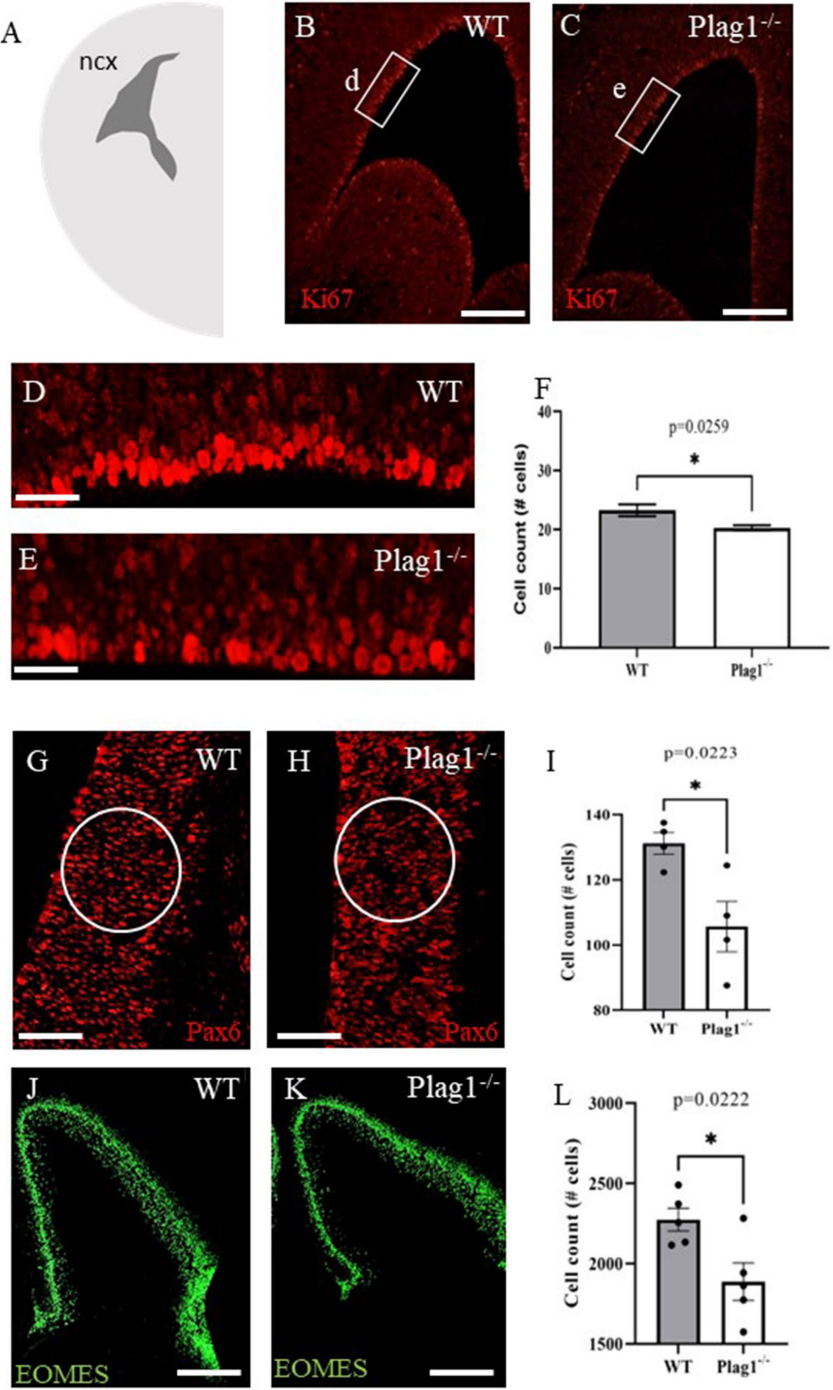
Deficiency of *Plag1* leads to reduced neocortical progenitor proliferation within the ventricular zone of the developing neocortex. Schematic representing the embryonic mouse brain at E14.5 (**A**; ncx, neocortex). Representative images of cell proliferation-marker Ki67 expression within the neocortex in male *Plag1* WT (**B**, **D**) and *Plag1*^−*/*−^ (**C**, **E**) at E14.5, showing significantly reduced cell numbers in the neocortex of *Plag1*^−*/*−^ embryos. Error bars in **F** represent the standard error of the mean (*n* = 5 per genotype), *p = 0.026 (unpaired *t*-test). Representative images of Pax6^+^ neural stem/progenitor cells in the developing cortex in male *Plag1* WT (**G**) and *Plag1*^−*/*−^ (**H**) E14.5 embryos. The white circle represents the regions analysed. Error bars in **I** represent the standard error of the mean, *p = 0.022 (unpaired *t*-test). Representative images of EOMES^+^ intermediate progenitor in the developing cortex of male *Plag1* WT (**J**) and *Plag1*^−*/*−^ (**K**) E14.5 embryos. Cell counts in **L** were performed on the entire field (equivalent hemisphere). Error bars represent the standard error of the mean, *p = 0.022 (unpaired *t*-test). Immunofluorescence images of Ki67, Pax6 and EOMES are representative of *n* = 5 individual animals, with a minimum of 3 sections analysed per animal. Scale bar = 200 μM (**B**, **C**, **J**, **K**), 50 μM (**G**, **H**) and 20 μM (**D**, **E**)

Given the striking layer-specific expression of *Plag1* in the cortex of adult brains, we investigated the role of *Plag1* during neurodevelopment. Previous studies had investigated *Plag1* function from embryonic day (E) 9.5 (E9.5) to E12.5 [17, 18], although these timepoints do not encompass the peak time-period of proliferative neural activity (E14.5) [27]. Following *Plag1* overexpression via electroporation into E12.5 neocortical progenitors, neuronal differentiation was inhibited, with cells retaining an immature phenotype [17]. In contrast, shRNA-mediated inhibition of *Plag1* also resulted in an inhibition of differentiation [18], suggesting that these transient modulatory approaches may not fully characterise the biological role of *Plag1*. Therefore, in order to determine the consequences of PLAG1 function during the highly proliferative phase of cortical development (E14.5), we examined the proliferative neocortical progenitors within the ventricular zone (VZ). Our data showed that loss of *Plag1* led to reduced neocortical progenitor proliferation within the ventricular zone of the developing neocortex (Fig. 4A). Immunohistochemical staining and quantification with the cell proliferation marker Ki67 revealed that *Plag1*^−*/*−^ mice presented with fewer proliferating cells in regions of the VZ, compared to WT mice (WT, 23.28 ± 0.99; *Plag1*^−*/*−^ 20.29 ± 0.46; *p* = 0.0259; Fig. 3B–F). Next, we determined whether reduced proliferation correlated with impaired production of mature cell types within this layer in *Plag1*^−*/*−^ mice. We found a significant decrease in the number of neocortical progenitors (*Pax6*^+^) in *Plag1*^−*/*−^ brains (105.7 ± 7.66) compared to WT littermates (131.2 ± 3.32; *p* = 0.0223; Fig. 4G–I). This was also true for EOMES+ intermediate progenitors (*Plag1*^−*/*−^, 1888 ± 116.5; WT, 2274 ± 71.2; *p* = 0.0222; Fig. 4J–L), indicating overall reduced (normalised) cellularity in the brains of mice lacking *Plag1*. Together, these data show an overall decreased abundance of proliferating and differentiating NSPCs within the *Plag1*^*−/−*^ mouse brain.

### Plag1 Deficiency Does Not Impact the Neurogenic Capacity of Neural Stem/Progenitor Cells Ex Vivo

**Fig. 5:**
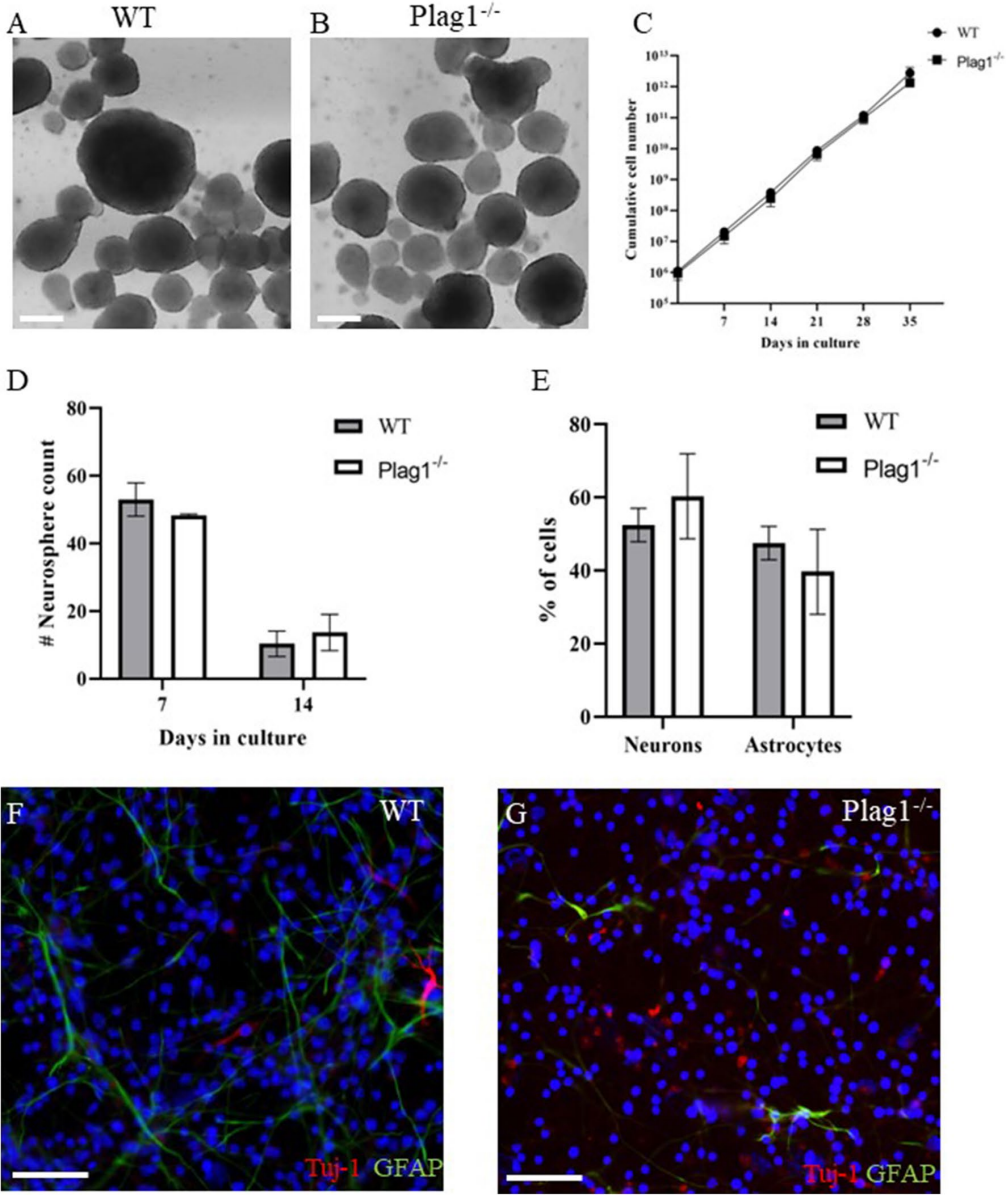
*Plag1* deficiency does not impact the neurogenic capacity or differentiation of NSPCs. The number of NSPCs in WT (**A**) and *Plag1*^−*/*−^ (**B**) neurospheres cultured at high density (1–5 × 10^5^ cells/mL) was not significantly different over 5 weeks *ex vivo* culture (**C**). Similarly, when cultured at a lower density (20–200 cells/ml), there was no difference in cell growth at 7 days or survival at 14 days (**D**). The potential of *Plag1*^−*/*−^ cells to differentiate into neurons and astrocytes was not significantly different to WT cells (**E**). Representative images of differentiated NSPCs in WT (**F**) and *Plag1*^−*/*−^ (**G**) cultures; *n* = 3 separate animals per genotype, experiment performed in duplicate. Scale bar = 50 μM (**A**, **B**) or 1000 μM (**F**, **G**)

To determine whether the reduction in NSPCs *in vivo* was due to cell-autonomous defects in the neurogenic capacity of *Plag1*^*−/−*^ neural progenitors, NSPCs were isolated from the lateral and medial ganglionic eminences of *Plag1*^*−/−*^ and WT embryos at E14.5 and cultured *ex vivo.* We found that there were no qualitative differences in the appearance of neurospheres formed from either WT or *Plag1*^*−/−*^ mouse brains (Fig. 5A, B). In order to determine whether there was a self-renewal defect in neurospheres derived from NSPCs lacking *Plag1* following prolonged ex-vivo culture (to deplete the NSPC pool), we cultured the neurospheres for a period of 35 days (5 weeks), passaging as appropriate [27]. Cumulative cell counts performed weekly indicated no difference in total cell production (i.e. neurogenic capacity) of *Plag1*^*−/−*^ (2.7 × 10^12^) cells compared to WT cells (1.4 × 10^12^; Fig. 5C) at the end of the 35-day culture period. Similarly, when we cultured *Plag1*^*−/−*^ and WT NSPCs at medium-density (20–200 starting cells), there was no difference in the number of viable neurospheres formed from *Plag1*^*−/−*^ compared to WT cultures at either 7D (WT 53.41 ± 4.17; *Plag1*^*−/−*^ 49.18 ± 0.89) or 14 d (WT 10.33 ± 3.76; *Plag1*^*−/−*^ 13.67 ± 5.36) (Fig. 5D).

High or medium-density cell cultures can form an “*in-vitro* niche” creating an environment with cell-to-cell contact and paracrine signalling [27, 29]. Therefore, to assess autonomous cellular behaviour independently of this “niche”, we cultured NSPCs at single-cell density in Terasaki wells, to determine whether the NSPCs from *Plag1*^*−/−*^ embryonic brains were able to maintain their self-renewal capacity in the absence of paracrine signalling. We found that the percentage of wells containing neurospheres comprising > 8-cells following 7-days culture (indicative of successful proliferation) was not significantly different in NSPCs derived from *Plag1*^*−/−*^ embryonic brains (63.06 ± 16.7%) relative to NSPCs derived from WT brains (71.04 ± 4.19%; Fig. S1A-B). Lastly, to determine whether *Plag1*^*−/−*^ NSPCs were able to differentiate into both neurons and astroglial cells, WT and *Plag1*^*−/−*^ neurospheres were induced to differentiate by treatment with 5% foetal calf serum. We found that the proportion of both neurons (β-tubulin^+^) and astrocytes (GFAP^+^) formed from *Plag1*^*−/−*^ NSPCs (neurons, 60.3 ± 11.6%; astrocytes 39.7 ± 11.6%) did not vary significantly from WT cells (neurons, 52.5 ± 4.58%; astrocytes 47.5 ± 4.58%), indicating that *ex vivo*, NSPCs can differentiate normally in the absence of *Plag1* (Fig. 5). Taken together, these surprising results suggest that loss of *Plag1* does not influence the growth, survival, self-renewal or differentiation of NSPCs *ex vivo.*

### Plag1 Deficiency Largely Does Not Alter Expression of Predicted Neural Target Genes Within the Developing Cortex

To address the lack of known gene targets of PLAG1, we utilised the transcription factor-target gene interaction database *TFLink* [19] to identify possible PLAG1 targets that may function in brain [30–32]. Next, we cross-referenced this list with some of the top-ranked differentially regulated genes identified previously by RNA-seq from E11.5 NSPCs, in which the expression of PLAG1 was inhibited by shRNA [18]. Of 47 differentially-regulated genes, 29 contained the putative Plag1-binding motif of a core GA/GGGC sequence followed by 6–10 nucleotides and then at least three guanine nucleotides. We selected five of these genes (with known neural functions) to examine in the cortex of E14.5 *Plag1*^*−/−*^ mice, alongside three other putative target genes involved in neurogenesis, namely *Dlx1* (a critical gene for neuronal differentiation and survival; [33]), *Ldb1* (involved in neuronal patterning, migration and differentiation; [34]) and *Ngn2* (a gene that drives neuronal differentiation from progenitor cells; [35]). Importantly, both *Dlx1* and *Ngn* 2 had been previously used as markers to demarcate regions of the developing telencephalon in *Plag1*^*−/−*^ embryonic telencephalon [17]. These latter genes were selected as they are specifically involved in neuronal differentiation and would likely not have been discovered in RNA-SEQ datasets from actively proliferating E11.5 NSPCs.

**Fig. 6:**
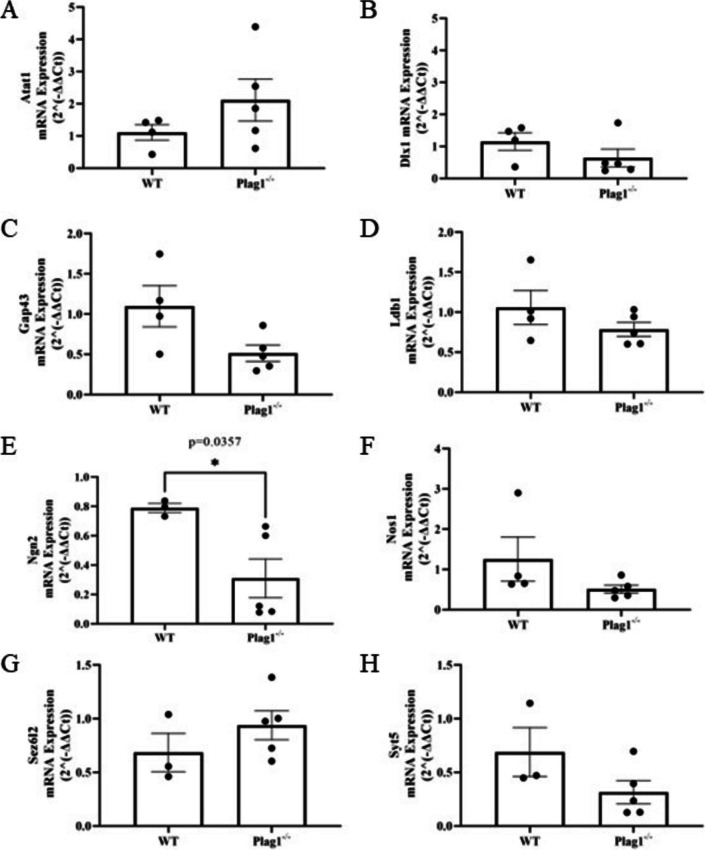
mRNA expression levels of putative neural target genes in the cortices of wild-type and *Plag1*^−*/*−^ mice. *Atat1*, alpha tubulin acetyltransferase 1; *Dlx1*, distal-less homeobox; *Gap43*, growth associated protein 43; *Ldb1*, LIM domain binding 1; *Ngn2*, Neurogenin 2; *Nos1*, nitric oxide synthase 1; *Sez6l2*, seizure-related homolog like 2; *Syt5*, synaptotagmin 5*.* Of these, only the expression of Ngn2 was significantly downregulated in *Plag1*^−*/*−^ mice (*p = 0.04; unpaired *t*-test; minimum *n* = 3 individual animals per genotype)

We examined the expression of these genes by qPCR and found that the expression of 7 out of 8 genes was not significantly different between the E14.5 cortices of WT and *Plag1*^*−/−*^ mouse brains (Fig. 6). Interestingly, however, we identified a single dysregulated gene, *Neurogenin 2* (*Ngn2*), which contains a putative *Plag1* binding site at position 3:127,422,088–127,422,116 (GRC mm39) within the *Ngn2* promoter region, and was significantly downregulated in *Plag1*^*−/−*^ mice (*p* = 0.04).

These data further highlight the differences in experimental paradigms to determine the nature of *Plag1*-dependent transcriptional pathways in understanding NSPC proliferation and differentiation within the brain.

## Discussion

Data presented within this study reveal novel areas of PLAG1 expression within the adult brain, providing new insight into potential region-specific functions of PLAG1 in growth, maintenance, signalling or neuronal function within the adult brain. Within the embryonic brain, we found decreased neural stem/progenitor cells (NSPCs) and intermediate progenitor cell populations within the developing cortex, although a suite of neurosphere assays to investigate the self-renewal capacity of NSPCs found no evidence that loss of *Plag1* impacted the cell-intrinsic proliferative, differentiation, survival or self-renewal capacity of NSPCs *ex vivo*. Nonetheless, our study serves as an excellent basis for future behavioural analyses in mice lacking *Plag1* and forms a solid base to specifically investigate regional neuronal interactions and signalling between different areas of the brain.

### PLAG1 in the Hippocampus

PLAG1 expression was abundant, albeit not ubiquitous, within the hippocampus, showing spatially restricted patterns of expression. Specifically, expression was restricted to the stratum pyramidale and stratum radiatum of the CA1 region, with very little expression in CA2, CA3 and the DG. The stratum pyramidale contains pyramidal cells; these integrate both excitatory and inhibitory information to generate outputs that transmit processed information from the CA1 region to other cortical and subcortical areas, indicating a putative role in synaptic integration and processing for PLAG1 [36]. The stratum radiatum receives inputs from the CA3 region to influence the excitability and response of the CA1 pyramidal neurons to the spatial and temporal information provided by the CA3 region [37], and we noted PLAG1 expression was more scattered throughout this region. The CA1 region acts as the main output from the hippocampus to the rest of the cortex, relaying crucial spatial memory information [38, 39]. Therefore, our expression data suggest that PLAG1 may be involved in spatial memory processing. Although previous behavioural data from *Plag1*^*−/−*^ mice did not find significant impairment to working memory that analysis was drawn based primarily only on experimental data from the *Y*-maze test [15]. That particular test has drawbacks for assessing spatial memory if behaviours are not stereotypical; an example of this would be if the animal shows anxiety-induced avoidance behaviours. In this instance, a better methodology would be the Morris water maze, as this is considered a more sensitive test for detecting hippocampal dysfunction, given that performance is associated with long-term potentiation and *N*-methyl-d-aspartate (*NMDA*) receptor function [40, 41].

Although we observed only sparse PLAG1 expression in the dentate gyrus (DG) of the adult mouse brain, the number of neurons in the DG was significantly reduced in *Plag1*^*−/−*^ brains relative to WT controls (Fig. 1.), consistent with an overall reduction in brain size. However, our analyses indicated that the neurons in the DG of *Plag1*^*−/−*^ mice qualitatively appeared less tightly-clustered together. A possible interpretation here concerns the role of Neurogenin 2 (*Ngn2*), a master regulator of neurogenesis [42] and the only gene we examined by qRT-PCR that showed differential expression in the brains of *Plag1*^*−/−*^ mice. *Ngn2* is required for DG development, and mice that lack *Ngn2* present with fewer neurons in the dentate gyrus [43]. Moreover, NGN2 phosphorylation has been described to drive motor neuron specification [44], and thus, it is plausible that reduced neuronal function throughout the brains of *Plag1*^*−/−*^ mice, particularly in areas of strong PLAG1 expression, may be correlated with reduced functional expression of NGN2. Future studies should focus on single-cell RNA-sequencing (scRNA-SEQ) approaches within the *Plag1*^*−/−*^ brain, particularly within the DG and more broadly the hippocampus proper, to test this hypothesis and investigate and correlate regional-specific expression of *Ngn2* within the spectrum of *Plag1*^+^ neural cells.

### PLAG1 Expression in the Choroid Plexus, Ependymal Cells, Sub-commissural Organ, Habenulae and Amygdala

The sub-commissural organ (SCO) is largely thought to mediate cerebro-spinal fluid (CSF) homeostasis; however, intriguing new evidence suggests it is also capable of influencing neurogenesis [45]. In addition to the NSPC marker *Pax6*, expression of the proliferation marker proliferating cell nuclear antigen (PCNA) has also been detected in the SCO in the adult brain, indicating that it is indeed an area of neural cell proliferation, consistent with the formation of new neurons [45]. Although PCNA had been detected within that region, the marker of active proliferation (the G_2_M phase of the cell cycle) ki67 was not detected, suggesting that under homeostatic conditions the SCO may remain quiescent, however may retain the potential for proliferation if needed [45]. Considering the well-established mitogenic functions associated with *Plag1*, we hypothesise that the pronounced expression of PLAG1 in the SCO may be involved in the neurogenic or proliferative potential within this region. Future studies to interrogate this theory should investigate proliferation and regeneration capabilities of NSPCs in these regions following *in vivo* neural insult (such as focal trauma, chemo/radiotherapy, neurotoxin administration or laser-ablation), to induce neurogenesis. Non-mammalian models with superior neural regeneration capabilities, such as the zebrafish, would also make a useful counterpoint to determine conserved roles of PLAG1 in neural proliferation.

Within the choroid plexus, we noted strongest expression within the ventral ependymal cells. These are multiciliated neuroepithelial cells of variable subtypes, classified by morphology and function [46]. The consistent expression of PLAG1 in these choroid plexus ependymal cells and the SCO suggests a putative role in CSF production [47]. Although Plag1 loss does not appear to be detrimental to CSF production, future approaches should determine whether the volume or composition of the CSF is defective in the brains and spinal cords of *Plag1*^*−/−*^ mice.

Our previous work characterising behavioural phenotypes of *Plag1*^*−/−*^ mice determined that these mice presented with a decrease in freezing behaviour and startle response [15]. This suggested the hypothesis that a dysfunctional amygdala may explain the reduced freezing behaviour in *Plag1*^*−/−*^ mice during the cued fear conditioning test. Consistent with this hypothesis, we noted strong expression of PLAG1 in the amygdala which was restricted to the basolateral amygdala nucleus (BLA) and the cortical amygdala area. The basolateral amygdala is well known for its role in fear conditioning and memory consolidation. Changes to normal BLA function would impair fear conditioning and similarly to previous *Plag1*^*−/−*^ behavioural results, would reduce freezing behaviour [48, 49].

More recent studies have also indicated a role of the habenula in fear conditioning, where pharmacological and optogenetic tools were used to manipulate the lateral habenula during fear conditioning testing. The results showed that interfering with the neuronal activity of the habenula during fear conditioning learning is altered [50]. Moreover, as the habenula receives input from the amygdala, this network may also influence fear conditioning [50]. The extensive *Plag1* expression we detected within both the habenula and amygdala leads us to speculate that PLAG1 may be a novel factor in the learning or habituation of certain fear responses.

Future approaches combining multiple behavioural test administration, coupled with neuronal tract tracing to determine whether any connections between the amygdala and habenula are altered in *Plag1*^*−/−*^ mice are clearly indicated, in order to uncover amygdala-habenula circuitry-related defects.

### PLAG1 in the Cortex

Cortical layers develop in an “inside-out” pattern, whereby layer 6 is formed first and layer 1 is the layer that is last-formed. The different layers of the cerebral cortex are distinguishable by their respective cytoarchitecture, and within these regions, PLAG1 expression showed strict regionalisation. In the primary somatosensory cortical layers, abundant expression can be seen in layers 1, 2/3, 4 and 6 whereas layer 5 presented with only sparse PLAG1 expression. Layer 5 (and 6) neurons integrate cortical and extracortical synaptic inputs and represent the primary output of the cerebral cortex. Layer 5 neurons also relay information back to the pons, tectum, brainstem spinal cord and striatum [51]; our data therefore are not strongly supportive of a role for PLAG1 in these layer 5-dependent processes; however, they do suggest putative roles in neuronal functions within other layers, which we outline here. The cortical layers are primarily made up of excitatory neurons that originate from the radial glial cell (RGC) pool from the ventricular zone (VZ) of the developing neocortex [52, 53]. Layers 2/3 integrate synaptic inputs from several brain areas and project this information throughout the cerebral cortex [54]. Layer 2/3 excitatory neurons comprise the major source of callosal projections, meaning that they relay information between hemispheres [55]. Similarly to layer 2/3, layer 4 also projects intracortically and represents the primary thalamo-recipient layer [56]. Layer 6 neurons share connections with layers 4 and 5 whilst receiving inhibitory information from adjacent neurons with layer 6 [57]. Extra-cortical projections arising from layer 6 target the thalamus [58], suggesting that PLAG1 may be involved in signalling between these regions. Together our expression data are clearly supportive for spatio-temporal regulation of *Plag1* within the cortex and are consistent with a putative role in intracortical and thalamic synaptic integration. However, our results do not support a role for *Plag1* in cortical output into pons, tectum brainstem, spinal cord or striatum. As per the neuronal tract tracing circuity experiments suggested for the amygdala-habenula axis, these approaches to visualise potential errors of connectivity within the brain of *Plag1*^*−/−*^ mice would further clarify the role of *Plag1* in neuronal signalling and function.

### PLAG1 in the Embryonic Brain

Early during development, mammalian NSPCs primarily generate neurons; this will ultimately determine the number of neurons in the brain. Essentially, dysregulation in the number of NSPCs at this developmental timepoint would lead to significant functional and cognitive deficits in adulthood, as these cells are critical for neurocircuitry formation. In comparison to the adult brain, where PLAG1 is expressed in several brain regions, the majority of PLAG1 expression within the developing brain is found in the dorsal telencephalon, diencephalon and midbrain, with limited hindbrain expression [7]. At the peak of proliferation in the developing mouse brain (E14.5), we investigated proliferative levels in the VZ and determined that *Plag1*^*−/−*^ mice present with significantly reduced proliferation in this region. Furthermore, there were fewer NSPCs and intermediate progenitor cells (IPCs) present, relative to the reduced brain size of *Plag1*^*−/−*^ mice. Although we see significantly fewer NSPCs and IPCs in the *Plag1*^*−/−*^ mice, existing behavioural data did not detect any clear cognitive deficits in male and female adult mice [15]. Taken together the reduction in NSPCs and IPCs would predict an overall reduction neuronal number in adults.

Our *in vivo* data confirm that PLAG1 is crucial for proliferation in the developing brain. However, unexpectedly, we found that this was not the case when we analysed the *ex vivo* (cell-autonomous) role of *Plag1* in cultured neurospheres. Whilst we saw a significant decrease in cell proliferation in the VZ and in NSPCs and intermediate progenitor cell populations *in vivo* (Fig. 3), we did not see changes in the proliferative potential of *Plag1*^*−/−*^ NSPCs. In order to eliminate the possible protective effects of the “*in vitro* niche”, we cultured the cells at single-cell densities to ameliorate the paracrine signalling present in high-density cultures [27]. We found that bulk neurosphere culture did not lead to any defects in *Plag1*^*−/−*^ neurospheres, and similarly, the clonal self-renewal capacity of *Plag1*^*−/−*^ mice was also not altered in a single cell Terasaki array. This important finding suggests PLAG1 plays a non-cell autonomous role within the microenvironment during neural development. In support of this, PLAG1 is known to directly regulate growth factor IGF2 [20], which is a paracrine factor that influences cell proliferation non-autonomously, both on its own and via insulin growth factor 1 receptor (*Igf1R*) activation and subsequent Ras/Raf/MAPK signalling [22].

Our data now further show dysregulation of *Ngn2*, a regulator of NSPC proliferation and neurogenesis. We therefore speculate that PLAG1 may work synergistically with multiple regulators of NSPC proliferation during brain development, and future studies should investigate possible effects of intercrossing mice comprising heterozygous and nullizygous deletions of the abovementioned genes with *Plag1*^*−/−*^ mice to determine potential synergistic effects and genetic haploinsufficiency on neurogenesis.

### The Neurogenic Role of Plag1—Comparison with Previous Studies

Several studies have now examined the role of *Plag1* in neurogenesis and proliferation, and it is interesting to compare and contrast these, owing to significant differences in methodology. In addition to morphogenic and molecular in vivo analyses of the brains of embryonic *Plag1*^*−/−*^ mice [17], two previously published approaches used transient methods to inhibit (shRNA) or over-express *Plag1* function in wild-type cells, either using retrovirus-mediated transduction [18] or in utero electroporation into the developing neocortex at E12.5 [17, 18].

Firstly, previous analysis of the *Plag1*^*−/−*^ embryonic mouse brain [17] showed a reduction in the proliferation of NPCs in the E12.5 cortex, consistent with the data we present here at E14.5. Although there is no difference in the presence of radial glial cells (*Pax6*^+^) or intermediate progenitors (*EOMES*/*Tbr2*^+^) at E12.5 [17], we show that there is a reduction in both these populations by E14.5 (Fig. 4J), suggesting that the reduced proliferation does ultimately impact on progenitor cell number as development proceeds—a novel finding. Similarly, transient inhibition of *Plag1* in vivo through in utero electroporation of *Plag1* shRNA at E11.5 [18] led to reduced *Tbr2*^+^ cell number by E13.5, suggesting that E12.5-E14.5 is the critical timepoint of *Plag1* function within the developing neocortex to drive neurogenesis.

In utero electroporation to drive in vivo *Plag1* over-expression in the E12.5 brain [17] led to a small but significant increase in proliferation 72 h after electroporation, alongside disrupted neocortical migration into the cortical plate and impaired neuronal differentiation. Conversely, in utero electroporation to drive in vivo *Plag1* over-expression in the E14.5 brain [18] led to increased neuronal differentiation, although the effect on proliferation was not examined. Similarly, over-expression of *Plag1* through retroviral-mediated transduction in E11.5 neurospheres cultured for both 3 and 9 days in vitro also increased neuronal differentiation [18], consistent with the above data. *Plag1* over-expression could also drive neuronal differentiation following retroviral transduction into E17.5 NSPCs, a timepoint when *Plag1* expression is normally reduced [18]. Together, these data highlight differing requirements for *Plag1* in neurogenesis depending on the stage of embryonic neural development. It is also highly likely that differences in factors such as overall mouse health, culture conditions, neurosphere density, passage number and growth factor supply could all lead to altered NSPC potential in vitro, and further explain differences in our findings.

Although methodological differences exist between studies, we would argue that the strength of our approach is the absence of cellular manipulations and transient inhibition, thereby representing the closest physiologically relevant model of human PLAG1 loss. Moreover, our data suggest that genetic compensation may also exist within *Plag1*^*−/−*^ mice, although previous studies argue this is not through upregulation of *Plag*-family members *Plagl1* or *Plagl2* [17]. Nonetheless, the differences apparent between neurosphere differentiation and gene expression seen between our genetic-deletion model and transient inhibition [18] strongly argue for the existence of genetic compensatory mechanisms.

### Plag1 Target Genes

There have only been small advances since the initial studies on *Plag1* target genes in 2004 [1]. One of the limitations with understanding more about *Plag1* target genes is that the consensus PLAG1 binding sequence is located in a significant number of locations within putative promoter and enhancer regions in the genome. In humans, 4739 genes contain a PLAG1 binding motif [19], representing ~25% of the human genome. Several PLAG1 target genes were reported as differentially regulated in *Plag1*^*−/−*^ mice NSPCs via bulk RNA-Seq [18]. However, these genes had not been further validated experimentally in other models, and target genes involved in neuronal differentiation [30–32] had not been described. Our gene expression analysis revealed no difference in expression levels of *Ata1*, *Gap43*, *Nos1*, *Sez6l2* and *Syt5*, illustrating the variability between the experimental approaches used. These studies highlight the molecular differences that exist following transient inhibition of PLAG1 compared to in vivo analysis of genetically-deficient cortical tissue, and again would argue for a degree of genetic compensation in *Plag1*^*−/−*^ mice.

From the several genes we examined, *Ngn2* was the only gene to be significantly downregulated in *Plag1*^*−/−*^ mice. The expression of *Ngn2* within the brain overall is well characterised [59, 60] and *Ngn2* function has been reported previously in several regions of strong *Plag1* expression identified here, including the cortex [61], dentate gyrus [43] and choroid plexus [62]. In fact *Ngn2* expression has been previously described (albeit not quantitated) within the dorsal telencephalic ventricular zone in *Plag1*^*−/−*^ mice at E12.5 [17]. We are at present unaware of any functional studies examining *Ngn2* within other regions of strong *Plag1* expression we report here, such as the basolateral amygdala, medial habenula or sub-commisural organ, and future studies analysing co-expression of PLAG1 and NGN2 within these areas, alongside scRNA-SEQ analyses, would better inform whether a true functional relationship exists between these genes during neuronal development in these regions.

Although expressed throughout corticogenesis, NGN2 is only required for early-born neurons to direct regional and glutamatergic phenotypic specificity common to all neocortical projection neurons, and subsequent laminar identity of deep-layer neurons [63, 64]. The reductions in NSPCs and IPCs discovered in *Plag1*^*−/−*^ cortices within the present study, therefore, are consistent with phenotypes seen following reduced *Ngn2* expression. Importantly, this would suggest that *Plag1* may influence cortical migration. Supporting this hypothesis is the fact that neurons fail to leave the ventricular/subventricular zone and migrate to the cortical plate in *Ngn2* KO mice [61]. Chromatin immunoprecipitation (ChIP) assays would confirm whether *Ngn2* was a true *Plag1* target within the developing cortex, and further lineage tracing experiments, whereby cells are electroporated and tracked via immunofluorescence, would further support our theory of a Neurogenin 2-dependent migration defect in *Plag1*^*−/−*^ cortices.

Taken together, our study comprehensively characterises the expression of PLAG1 within the adult brain, begins to correlate known behavioural defects with regionalised expression and argues against a cell-autonomous role for PLAG1 in NSPC proliferation. Future work should focus on further identification of *Plag1* target genes in the context of the adult and developing brain, through genome wide ChIP-SEQ approaches correlated with scRNA-SEQ analyses of precise regionalised cellular expression. These data could then be extended to better understand functional *Plag1*-dependent mechanistic relationships in neural development, NSPC proliferation and regeneration in vivo (especially following insult), novel behavioural phenotypes and potentially also age-related neurodegeneration.

### Supplementary Information

ESM 1 **Table S1**. Quantitative polymerase chain reaction (qPCR) primer sequences (JPG 68 kb)

ESM 2 **Fig. S1**. Self-renewal of *Plag1*^-/-^ NSPCs cultured at single-cell density. When cultured as single cells, there was no difference between genotypes for the percentage of wells containing 8+ cells over the time course (**A**). NSPC counts from single-cell culture in Terasaki wells, data from *n* = 3 plates per genotype, experiment performed in duplicate. Each cell represents the number of wells that contained the corresponding number of cells (from 0 cells to more than 8 cells in the individual well; **B**). (JPG 84 kb)

## Data Availability

All data supporting the conclusions in this study will be made available on request. Where feasible and allowable under existing Material Transfer Agreements, any materials used in this project will be made available on request. The transcription factor-target gene interaction database *TFLink* can be accessed here: https://tflink.net/.
