# Supplementary figures and images for: The Role of PLAG1 in Mouse Brain Development and Neurogenesis
Source: Mol Neurobiol. 2024 Jan 19;61(8):5851–67. doi: 10.1007/s12035-024-03943-w (PMC11249490; doi:10.1007/s12035-024-03943-w)

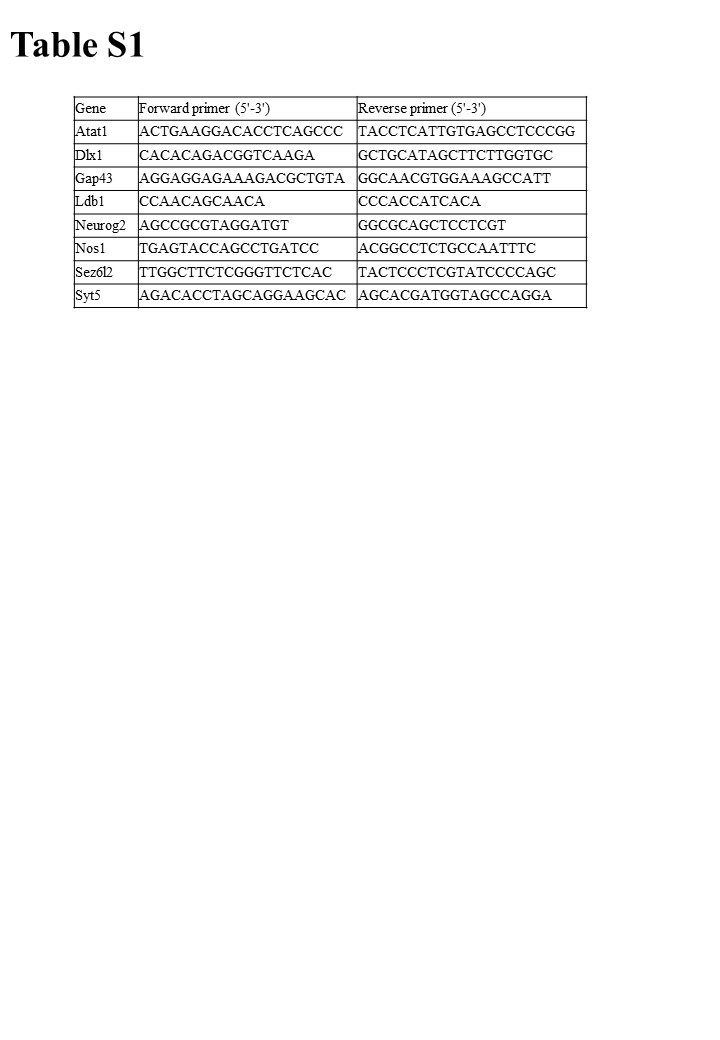

Supplement: Supplementary file 1 — Table S1. Quantitative polymerase chain reaction (qPCR) primer sequences (JPG 68 kb) [file 12035_2024_3943_MOESM1_ESM.jpg]

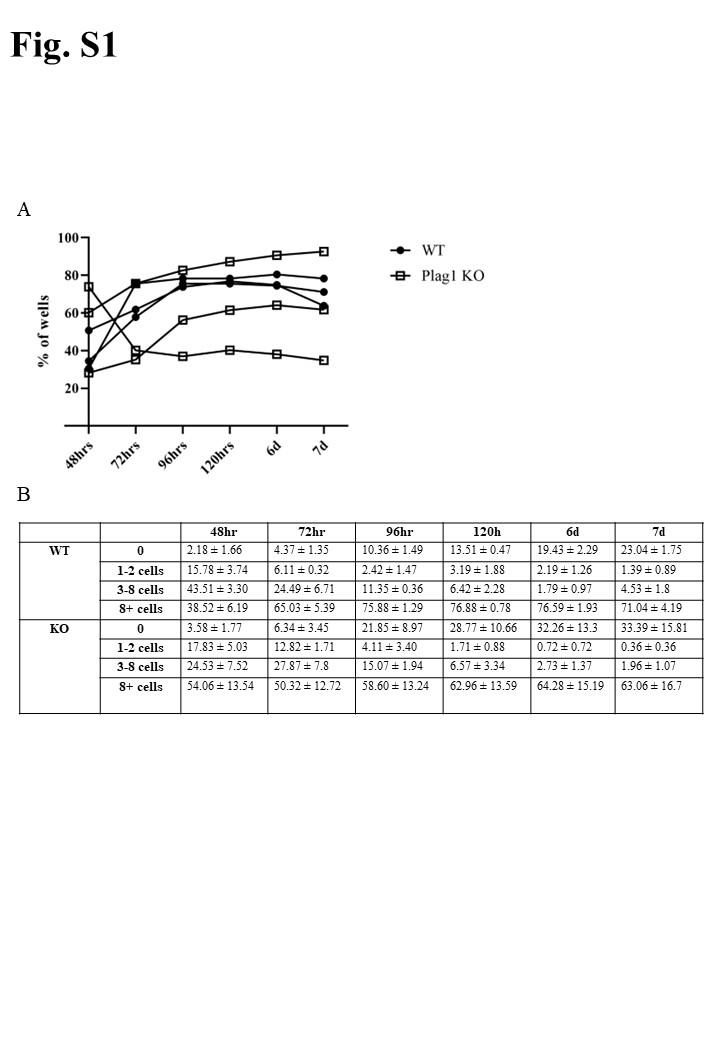

Supplement: Supplementary file 2 — Fig. S1. Self-renewal of Plag1-/- NSPCs cultured at single-cell density. When cultured as single cells, there was no difference between genotypes for the percentage of wells containing 8+ cells over the time course (A). NSPC counts from single-cell culture in Terasaki wells, data from n = 3 plates per genotype, experiment performed in duplicate. Each cell represents the number of wells that contained the corresponding number of cells (from 0 cells to more than 8 cells in the individual well; B). (JPG 84 kb) [file 12035_2024_3943_MOESM2_ESM.jpg]
